# Bacillus Calmette-Guérin–Related Mycotic Pseudoaneurysm in the Aortic Arch: Clinical and Operative Challenges

**DOI:** 10.1093/icvts/ivag120

**Published:** 2026-04-24

**Authors:** Sophie Bonni, Natasja W M Ramnath, Koen Selten, Ajay Moza

**Affiliations:** Department of Cardiac Surgery, RWTH Aachen University Hospital, Aachen 52074, Germany; Department of Cardiac Surgery, RWTH Aachen University Hospital, Aachen 52074, Germany; Department of Cardiac Surgery, RWTH Aachen University Hospital, Aachen 52074, Germany; Department of Cardiac Surgery, RWTH Aachen University Hospital, Aachen 52074, Germany

**Keywords:** mycotic aneurysm, Bacillus Calmette-Guérin, open surgical repair

## Abstract

Mycotic aortic aneurysms following intravesical Bacillus Calmette-Guérin therapy for urothelial carcinoma are rare but life-threatening.

A male patient with Bacillus Calmette-Guérin-treated bladder cancer presented with syncope, cough, and fever. Computed tomography showed a distal arch pseudoaneurysm, and positron emission tomography indicated an infection. Treatment included replacement of the aortic arch and the descending aorta and the use of antimicrobial agents. Histopathological analyses confirmed a *Mycobacterium bovis* infection. The patient died 1 month postoperatively of stroke and organ failure. Early recognition and multidisciplinary management are crucial.

**Clinical registration number:** Ethics Committee of the Medical Faculty of RWTH Aachen (EK 25-341).

## BACKGROUND

Bacillus Calmette-Guérin (BCG), an attenuated strain of *Mycobacterium bovis*, is administered intravesically to stimulate immune responses in patients with non-muscle-invasive bladder cancer.^1^ An *M. bovis* infection can cause aortic ulcers, vascular graft infections, or ruptured aneurysms.^2^ Although BCG primarily affects the abdominal aorta, peripheral arteries may be involved.^3^ We present a rare case of an infected pseudoaneurysm of the distal aortic arch and descending aorta after BCG therapy, highlighting the need for early recognition and multidisciplinary management of this complication.

## CASE REPORT

A 78-year-old man was initially admitted to an external hospital after experiencing orthostatic syncope and a 3-week history of fever and persistent cough. Examination results were unremarkable. Inflammatory markers were elevated.

The medical history included BCG therapy for urothelial carcinoma, chronic bronchitis, Barrett’s oesophagus, gastritis, and a pacemaker for sick sinus syndrome.

The initial work-up targeted common infections. Chest radiography showed emphysematous changes without consolidation, and *Klebsiella pneumoniae* grew in a urine culture. Because there was no clear focus, empirical ampicillin/sulbactam was initiated. Serial blood cultures remained consistently negative for any pathogenic growth.

**Figure 1.**
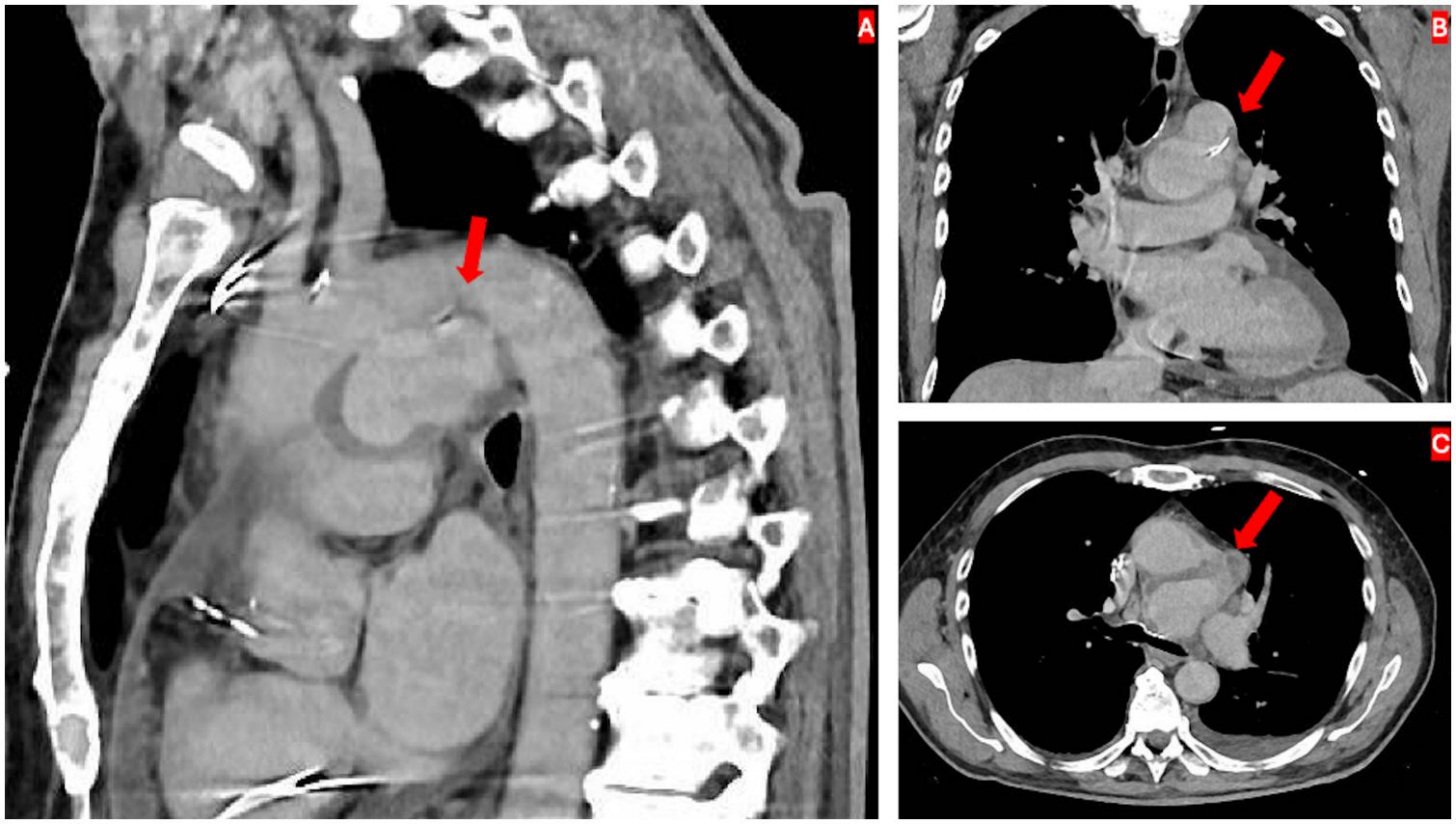
Sagittal (A), Coronal (B), and Transverse (C) Sections of Preoperative Contrast-Enhanced Computed Tomography Showing a Distal Arch Pseudoaneurysm from a Penetrating Aortic Ulcer Measuring 4.5 × 7.2 × 3.7 cm

**Figure 2.**
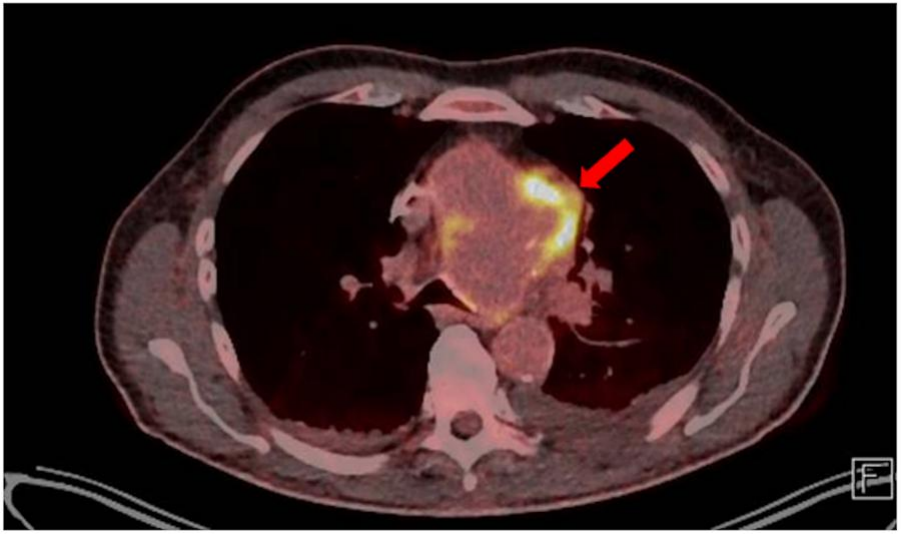
Transverse Section of Positron Emission Tomography Showing High Metabolic Activity in the Aortic Arch, Confirming an Infected Pseudoaneurysm

Due to persistent fever, contrast-enhanced computed tomography (CT) was performed, revealing a 4.5 × 7.2 × 3.7 cm pseudoaneurysm arising from a penetrating aortic ulcer in the aortic arch (Figure 1). Positron emission tomography (PET) revealed intense tracer uptake at the pseudoaneurysm, indicating infection (Figure 2).

Upon transfer of the patient to our clinic, broad-spectrum therapy was escalated to piperacillin/tazobactam and vancomycin. After the patient developed acute renal failure, vancomycin was switched to linezolid.

An operation was performed because of the risk of rupture. Cardiopulmonary bypass was established via femoral venous and ascending aortic cannulations. With the patient under deep hypothermic arrest through a left lateral thoracotomy, the aortic arch and descending thoracic aorta were replaced with a 26-mm tube graft. The bypass time was 218 min, the cross-clamp time was 104 min, and the circulatory arrest time was 84 min.

Postoperative complications included a left middle cerebral artery stroke requiring a thrombectomy and stenting and multi-organ failure.

Intraoperative samples were positive for *M. bovis* DNA. Triple antituberculosis therapy was initiated; however, the patient died 1 month postoperatively.

## DISCUSSION

This case highlights challenges in management of infections of the aortic arch. Patients present with nonspecific symptoms like fever, weight loss, or back pain.^3^ Our patient had persistent cough and fever, which delayed medical attention. Informing patients undergoing BCG therapy about rare complications may increase awareness and facilitate earlier evaluations. Microbiological confirmation is challenging in the presence of prior antibiotic use or atypical organisms. Initial test results were inconclusive. After a history of previous BCG administration, more specific early diagnostic evaluation of mycobacterial cultures or PCR testing could have been pursued. Emerging diagnostic tools, such as microbial cell-free DNA sequencing, can aid in early diagnoses.^4^ Moreover, early detection of pseudoaneurysms requires suspicion and timely imaging. In this case, contrast-enhanced CT followed only after persistent fever. In patients with systemic symptoms after BCG therapy or unexplained fever, early CT scans can anticipate diagnosis of infectious pseudoaneurysms. The PET-CT imaging showed intense uptake at the aortic arch, supporting the diagnosis of an infected pseudoaneurysm, aligning with evidence that PET-CT imaging improves detection of vascular infections when conventional imaging or microbiological testing results are inconclusive. Management of this situation involves antituberculosis therapy and an operation, including endovascular procedures, hybrid techniques, and open reconstructions. Reports support open repair for mycotic aortic lesions.^5^ Given the complexity and risk of recurrent infection with endovascular repair, we chose an open operation. Despite intensive management, the patient died 1 month postoperatively.

Prevention, early detection, and optimal treatment are complementary. Informing patients, early recognition with a low threshold for blood cultures and imaging, prompt tissue sampling, targeted therapy, and multidisciplinary involvement can limit the progression of infection and reduce the risk of large pseudoaneurysms or rupture.

## CONCLUSION

The distal aortic arch is rarely affected after BCG therapy, making this case particularly notable. We highlight the management of complex infections of the aortic arch. Early diagnosis and individualized treatment through collaboration are essential.

## Data Availability

The data underlying this article will be shared on reasonable request to the corresponding author.
